# Associations of Lipoprotein(a) Level with Cerebral Small Vessel Disease in Patients with Alzheimer’s Disease

**DOI:** 10.3390/brainsci14010034

**Published:** 2023-12-29

**Authors:** Nihong Chen, Fuping Jiang, Xiangliang Chen, Lin Zhu, Na Qiao, Junshan Zhou, Yingdong Zhang

**Affiliations:** 1 Department of Neurology, Nanjing First Hospital, Nanjing Medical University, Nanjing 210006, China; neon_2000@163.com (N.C.); chenxl@njmu.edu.cn (X.C.); julinzz@163.com (L.Z.); zhjsh333@126.com (J.Z.); 2 Department of Neurology, Nanjing Yuhua Hospital, Yuhua Branch of Nanjing First Hospital, Nanjing 210039, China; 18238831198@163.com; 3 Department of Geriatrics, Nanjing First Hospital, Nanjing Medical University, Nanjing 210006, China; fupingjiang@163.com

**Keywords:** lipoprotein(a), cerebral small vessel disease, Alzheimer’s disease

## Abstract

Background: This study aimed to examine the association of lipoprotein(a) [Lp(a)] level with the burden of cerebral small vessel disease (CSVD) in patients with Alzheimer’s disease (AD). Methods: Data from 111 consecutive patients with AD admitted to Nanjing First Hospital from 2015 to 2022 were retrospectively analyzed in this study. Serum Lp(a) concentrations were grouped into tertiles (T1–T3). Brain magnetic resonance imaging (MRI) was rated for the presence of CSVD, including enlarged perivascular spaces (EPVS), lacunes, white-matter lesions, and cerebral microbleeds (CMBs). The CSVD burden was calculated by summing the scores of each MRI marker at baseline. A binary or ordinal logistic regression model was used to estimate the relationship of serum Lp(a) levels with CSVD burden and each MRI marker. Results: Patients with higher tertiles of Lp(a) levels were less likely to have any CSVD (T1, 94.6%; T2, 78.4%; T3, 66.2%; *p* = 0.013). Multivariable analysis found that Lp(a) levels were inversely associated with the presence of CSVD (T2 vs. T1: adjusted odds ratio [aOR] 0.132, 95% confidence interval [CI] 0.018–0.946, *p* = 0.044; T3 vs. T1: aOR 0.109, 95% CI 0.016–0.737, *p* = 0.023) and CSVD burden (T3 vs. T1: aOR 0.576, 95% CI 0.362–0.915, *p* = 0.019). The independent relationship between Lp(a) levels and individual CSVD features was significant for moderate-to-severe EPVS in the centrum semiovale (T2 vs. T1: aOR 0.059, 95% CI 0.006–0.542, *p* = 0.012; T3 vs. T1: aOR 0.029, 95% CI 0.003–0.273, *p* = 0.002) and CMBs (T3 vs. T1: aOR 0.144, 95% CI 0.029–0.716, *p* = 0.018). Conclusions: In this study, serum Lp(a) level was inversely associated with CSVD in AD patients.

## 1. Introduction

Lipoprotein(a) [Lp(a)] is a low-density lipoprotein (LDL) particle with its apolipoprotein B-100 (apoB100) covalently linked to the apolipoprotein(a). Although the physiological function of Lp(a) remains unclear, it is currently recognized as a causal risk factor for atherosclerotic cardiovascular diseases [1,2,3,4]. Cerebral small vessel disease (CSVD) is characterized by pathological changes in cerebral arterioles, venules and capillaries, with clinical and imaging evidence [5]. Recent studies have found that different from the relationship between serum Lp(a) level and atherosclerosis of the large arteries, the serum Lp(a) level is negatively correlated with small vessel stroke as well as CSVD and its total burden [6,7].

Alzheimer’s disease (AD) is the most prevalent cause of dementia in the elderly, accounting for approximately 60% of all dementia cases [8]. Neuropathological studies have found that 79.9% of AD patients have vascular pathological changes, among whom 40.8% have cerebral amyloid angiopathy (CAA) and 39.8% have age-related atherosclerosis [9]. The abnormal function of cerebral small vessels could aggravate the deposition of amyloid beta (Aβ) in the brain [10,11]. It is suggested that CSVD and AD are in a reciprocal relationship and the former plays an important role in the occurrence and development of AD. Previous studies have demonstrated that high Lp(a) levels are associated with an increased occurrence of vascular dementia (VD) [12,13] but a reduced risk of Alzheimer’s disease (AD) [12]. This indicates that there may be a protective effect of Lp(a) on CSVD in AD patients.

To the best of our knowledge, few studies to date have investigated the association between serum Lp(a) level and CSVD in AD patients. Hence, we retrospectively conducted a cross-sectional study of AD patients admitted to our hospital in the past seven years to clarify this relationship.

## 2. Materials and Methods

### 2.1. Study Population

This study was approved by the Ethics Committee of Nanjing First Hospital, Nanjing Medical University, with the approval number 20211011-05, and it was conducted in accordance with the Declaration of Helsinki.

In this study, we retrospectively reviewed data from 111 consecutive AD patients admitted to the Department of Neurology in Nanjing First Hospital from January 2015 to December 2022. All the participants met the criteria for probable AD dementia according to the National Institute on Aging–Alzheimer’s Association (NIA-AA) guideline [14].

Exclusion criteria were: (1) vascular dementia, (2) dementia caused by other degenerative causes, such as frontotemporal dementia, dementia with Lewy bodies, Pick’s disease, etc., (3) history of traumatic brain injury or malignant neoplasms, (4) chronic renal failure, (5) in the acute stage of diseases, and (6) insufficient data on Lp(a) or CSVD markers.

### 2.2. Data Collection and Measurement

Cardiovascular risk profiling, including basic information of age and sex, and the history of hypertension, hyperlipidemia, diabetes, atrial fibrillation, coronary heart disease, smoking, and drinking, was performed as a clinical routine for all the patients. Blood samples were drawn after overnight fasting for the measurement of serum Lp(a), glucose, total cholesterol (TC), low-density lipoprotein cholesterol (LDL-C), high-density lipoprotein cholesterol (HDL-C), triglyceride (TG), serum creatinine (Scr), blood urea nitrogen (BUN), and glycated hemoglobin (HbA1c). Among them, serum Lp(a) concentrations were measured using an immunoturbidimetry assay, with a range of 10–3200 mg/L. All the testing was conducted within 4 h of blood sampling at the central laboratory in Nanjing First Hospital by technicians who were blind to the clinical information.

### 2.3. MRI Acquisition and Assessment

All eligible patients underwent a brain magnetic resonance imaging (MRI) using a 3.0 T scanner ((Ingenia, Philips Medical Systems) with an eight-channel receiver array head coil. The imaging protocol included T2-weighted images (repetition time [TR]/echo time [TE], 7000/120 ms; section thickness, 6 mm; matrix, 230 × 230), fluid-attenuated inversion recovery (FLAIR) images (TR/TE, 7000/120 ms; section thickness, 6 mm; matrix, 356 × 151), DWI images (TR/TE, 2501/98 ms; section thickness, 6 mm; matrix, 152 × 122; DWI was obtained with b values of 0 and 1000 s/mm^2^) and SWI images (TR/TE, 22/34 ms; section thickness, 0.5 mm; matrix, 276 × 319).

All imaging data were collected during routine clinical practice in the Department of Radiology at Nanjing First Hospital.

Four neuroimaging markers of CSVD—that is, enlarged perivascular spaces (EPVS), white-matter hyperintensities (WMHs), lacunes, and cerebral microbleeds (CMBs)—were graded according to the STandards for ReportIng Vascular changes on nEuroimaging criteria [15].

EPVS were defined as >1 mm in diameter, cerebrospinal fluid-isointense lesions along the penetrating arteries on axial T2-weighted MRIs. The presence of EPVS in the basal ganglia (BG-EPVS) and centrum semiovale (CSO-EPVS) was visually assessed according to the rating scale by Potter, Morris and Wardlaw (https://www.ed.ac.uk/files/imports/fileManager/epvs-rating-scale-user-guide.pdf, accessed on 26 December 2023) and was graded as 0 = none, 1 = 1–10, 2 = 11–20, 3 = 21–40 and 4 ≥ 40 EPVS, according to a previously validated protocol [16].

The severity of WMHs was rated according to the Fazekas scale [17] on FLAIR images. The periventricular WMHs were rated as 0 = absent, 1 = “caps” or pencil-thin lining, 2 = smooth “halo”, and 3 = irregular periventricular signal extending into the deep white matter. The deep WMHs were rated as 0 = absent, 1 = punctate foci, 2 = beginning confluence, and 3 = large confluent areas.

Lacunes were defined as hyperintense lesions in the subcortical, basal ganglia or brainstem areas, with a diameter of 3–15 mm on T2-weighted images without any increased signal on DWI.

CMBs were defined as small (less than 10 mm in diameter), rounded, hypodense lesions within brain parenchyma on the SWI images [18].

The total CSVD score, also called the CSVD burden, was rated on a scale of 0 to 4, by allocating 1 point to BG-EPVS > 10, 1 point to the presence of lacunes, 1 point to confluent WMHs (i.e., periventricular Fazekas 3 or deep Fazekas 2–3), and 1 point to the presence of CMBs. The presence of CSVD was defined as patients with a total CSVD score ≥1 point [19].

Imaging data were assessed by two trained neurologists who were blinded to the clinical information. Inconsistencies were determined by another reader. Good interobserver reproducibility was found for each CSVD marker between raters (kappa = 0.90 for EPVS, 0.82 for WMHs, 0.80 for lacunes, and 0.80 for CMBs, respectively).

### 2.4. Statistical Analysis

Categorical variables are presented as frequency with proportion, and continuous variables as mean ± standard deviation (SD) or median with interquartile range (IQR) for normal and non-normal distribution, as appropriate. Comparisons of baseline characteristics among Lp(a) tertile groups were performed by one-way analysis of variance or Kruskal–Wallis test for continuous variables, and Pearson’s χ2 tests or Fisher exact tests for categorical variables. Multivariable binary and ordinal logistic regression were used to examine the relationship of Lp(a) concentrations with the presence of CSVD and a shift in the direction of a higher CSVD burden, both using the first tertile as the reference. The odds ratio (OR) with a 95% confidence interval (CI) was calculated for each regression model. For each dependent variable, potential confounders were adjusted for in two models. Model 1 included age and sex only, whereas Model 2 was additionally adjusted for body mass index (BMI), current smoking, current drinking, histories of hypertension, diabetes, atrial fibrillation and coronary heart disease, as well as levels of TC, TG, LDL-C, and HDL-C.

Data are available to researchers on request by contacting the corresponding author. A two-sided *p* < 0.05 was considered to be statistically significant. All analyses were performed with SPSS 23.0 software (Armonk, NY, USA).

## 3. Results

### 3.1. Baseline Characteristics

A total of 111 subjects with a mean age of 75.27 ± 9.70 years and a male percentage of 39.6% were included in the present study. The demographic and basal clinical characteristics of patients stratified by tertiles of Lp(a) are presented in Table 1, with no statistically significant difference between groups, except that there was a trend of an increase in LDL-C concentrations towards higher Lp(a) tertiles, with borderline significance (*p* = 0.076).

### 3.2. Association of Lp(a) Levels with the Presence and Burden of CSVD

**Figure 1:**
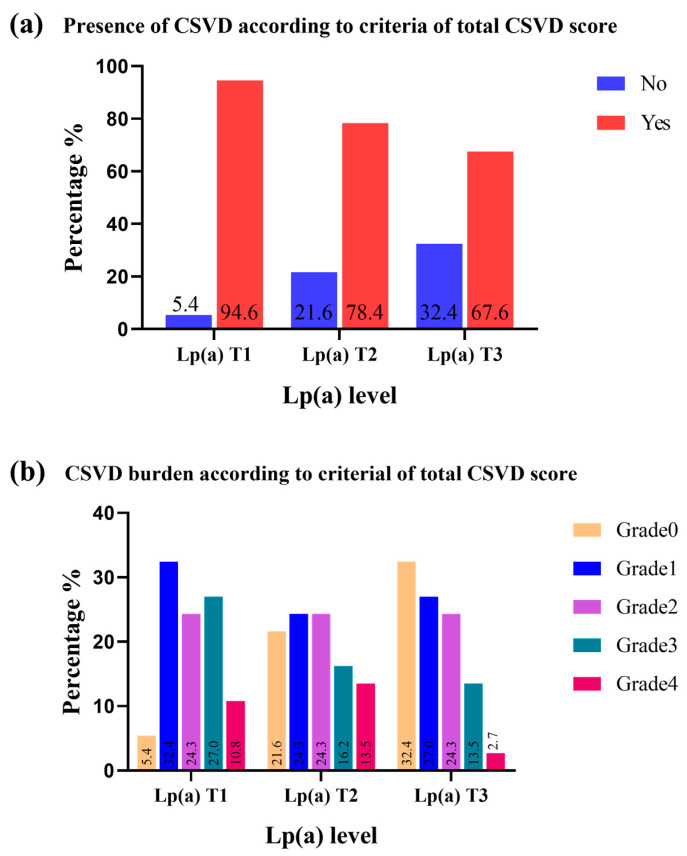
Prevalence of CSVD (**a**) and CSVD burden (**b**) in different Lp(a) levels.

There were 89 (80.2%) participants with CSVD in our cohort, among whom 69.4% had BG-PVS > 10, 42.3% had lacunes, 36.9% had periventricular WMHs extending into the deep white matter or had confluent deep WMHs, and 18.9% had CMBs. Of all the included subjects, 31 (27.9%), 27 (24.3%), 10 (9%) and 21 (18.9%) had CSVD burden scores of 1, 2, 3 and 4, respectively. The prevalence and burden of CSVD in Lp(a) tertiles are shown in Figure 1.

The association between Lp(a) and the presence of CSVD is shown in Table 2. After adjusting for age and sex (Model 1), subjects in the second and third tertiles of Lp(a) concentrations both had a lower risk of having any CSVD (OR 0.141, [95% CI, 0.023–0.841]; OR 0.116, [95% CI, 0.021–0.655]). This association remained after additional adjustment for BMI, current smoker, current drinker, hypertension, diabetes, history of atrial fibrillation and coronary heart disease, TC, TG, HDL-C, and LDL-C (Model 2).

Associations between tertiles of Lp(a) levels and CSVD burden based on ordinal logistic regression are shown in Table 3. Compared with patients in the first tertile of Lp(a) levels, those in the third tertile were associated with reduced odds of CSVD burden (Model 1: common OR [cOR], 0.501 [95% CI, 0.308–0.815]; model 2: cOR, 0.576 [95% CI, 0.362–0.915]).

The association of individual neuroimaging markers of CSVD with Lp(a) indicated that both the second and third tertiles of Lp(a) level were associated with a reduced odds of CSO-EPVS >10 (Model 2: cOR, 0.059 [95% CI, 0.006–0.542); cOR, 0.029 [95% CI, 0.003–0.273]). Meanwhile, patients in the third tertiles of Lp(a) levels were significantly associated with a decreased odds of CMBs (Model 2: cOR, 0.144 [95% CI, 0.029–0.716]). While patients with higher tertiles of Lp(a) level were less likely to be present with lacunes or confluent WMHs, both were not significant. The third tertiles of Lp(a) level were significantly associated with a reduced odds of BG-EPVS >10 (Model 1: cOR, 0.342 [95% CI, 0.119–0.980]), but when further corrected for cofounders, the significance no longer existed (Model 2: cOR, 0.530 [95% CI, 0.143–1.964]) (Table 4).

## 4. Discussion

In the present study, we retrospectively investigated the association between serum Lp(a) levels and the MRI markers of CSVD. We found that there was a significant negative correlation between serum Lp(a) levels and the risk of having any CSVD and the total burden of CSVD in AD patients. Specifically, higher Lp(a) concentrations were associated with less-severe EPVS in the centrum semiovale and a lower likelihood of CMBs in patients diagnosed with AD.

Previous studies found that a high Lp(a) level was related to carotid atherosclerosis [20,21] and atherothrombotic stroke [22] but not small vessel stroke [23,24]. A recent Mendelian randomization study showed an inverse association with small vessel stroke and Alzheimer’s disease [7]. Our study further demonstrated that an elevated serum Lp(a) level was related to low odds of the presence and burden of CSVD. This is concordant with the conclusion of a recent community population-based study by Yilong Wang et al., in which subjects with the third tertile of Lp(a) level had a decreased odds of the presence of CSVD (25.9% vs. 31.7%, adj.OR 0.74, 95% CI 0.60 to 0.92) and a lower CSVD burden (adj.cOR 0.76, 95% CI 0.62 to 0.94) [6]. However, our study showed an unexpectedly low OR value in the second and third tertiles of serum Lp(a) levels for the incidence of CSVD in the AD population when taking the first tertile as a reference. This may be due to the small number of patients without CSVD in our study population and the fact that there were only 2, 8 and 12 patients without CSVD with the lowest level of serum Lp(a) to the highest, respectively, resulting in a large difference in the ratio. Anyhow, it is suggested that the relationship between serum Lp(a) level and the occurrence and development of CSVD not only exists in the old community population but also is likely to exist in older AD patients. This correlation does not change with age and disease profile. Since serum Lp(a) concentration is relatively stable throughout a person’s life [25], and the occurrence and development of AD are closely related to CSVD, this persistent negative correlation between serum Lp(a) level and CSVD mentioned above may partially explain this protective effect of serum Lp(a) against AD.

In addition, there was a significant correlation between serum Lp(a) levels and individual CSVD features such as CSO-EPVS and CMBs. However, the significant negative association between Lp(a) levels and BG-EPVS was not significant after adjustment for confounders, and the negative trend of Lp(a) levels in correlation to the presence of WMH and lacune did not reach statical significance. However, in the above-mentioned large-scale cohort study, none of the neuroimaging markers of CSVD had a significant correlation with Lp(a) tertiles [6]. The reason may be due to a higher prevalence of people of an older age (75.3 vs. 61.2 years old) and AD profiles in our population, resulting in a significantly higher incidence of CSVD and various CSVD MRI markers. Furthermore, the concentration of serum Lp(a) in our study population was higher than that in the previous study (T1, <86 mg/L; T2, 86–183 mg/L; T3, ≥183 mg/L vs. T1, <40 mg/L; T2, 40–105 mg/L; T3, ≥106 mg/L), and the Lp(a) level was significantly different among the groups, so the negative correlation between serum Lp(a) level and CSVD MRI markers in each group could be better illustrated.

It is not yet clear why the significance of the relationship between Lp(a) and various MRI markers of CSVD was inconsistent in the AD population in our study. A study of 3976 brain specimens from patients older than 65 years [26] showed that with the increase in AD neuropathological changes, the proportion of moderate and severe CAA also gradually increased, suggesting a close relationship between AD and CAA. Previous studies have shown that EPVS in the basal ganglia are usually due to arteriolar sclerosis, while EPVS in the centrum hemi-oval are due to Aβ deposition [27,28], which suggested that CSO-EPVS in AD patients are mainly from CAA induced by Aβ deposition. The incidence of CMBs in CAA and AD patients was both significantly higher than that in healthy people and other dementia patients [29,30,31]. WMH and LI are common to arteriolar sclerosis and CAA. Our study shows that Lp(a) is more closely related to CSO-EPVS and CMBs, which seems to suggest that Lp(a) has inconsistent protective effects on CSVD of different etiologies and may have better protective effects on cerebrovascular amyloidosis. In addition, it has been reported that a high Lp(a) level appears to promote white-matter lesions and lacunes due to atherosclerosis and ischemia [12,32], which may counteract the protective effect on CSVD of Lp(a), resulting in an insignificant relationship between Lp(a) and WMH and lacunes in this study.

The underlying mechanism suggesting that elevated Lp(a) level was correlated with a lower risk of CSVD in AD patients is poorly understood. One possible explanation is that Lp(a) can enter the brain parenchyma and cerebrospinal fluid through the impaired blood–brain barrier [33], and Lp(a) can bind to a large number of oxidized phospholipids [34], which can greatly reduce brain inflammation if Lp(a) can be normally recycled. Lipoproteins, such as apoA-1, may be involved in the regulation of cholesterol metabolism in the cerebrospinal fluid [35]. It is suspected that Lp(a) may also be involved in the metabolism of lipoproteins in the brain and the maintenance of glioneurovascular units [36]. As reported by Moosers et al. [37], apoprotein(a) might be involved in lipoprotein metabolism within the brain. Although the majority of Lp(a) is synthesized in the liver, apoprotein(a) mRNA is identified in the rhesus brain. This implies the possibility that, if Lp(a) were produced in the brain under certain conditions, Lp(a) can combine with apoE to form apoE-enriched Lp(a) [38], which has a greater affinity for heparan sulfate proteoglycans(HSPG) than lipoprotein(a) particles without apoE, thus accelerating the non-receptor clearance of apoE [39]. Studies also suggested that apo(a) or Lp(a) could bind to apoE or compete with apoE receptors [40], resulting in the acceleration of the dissociation of apoE4 from the receptor, thereby speeding up the turnover of cholesterol and improving the function of brain cells and small blood vessels. Moreover, a previous study showed that APOE ε2 and APOE ε4 were associated with MRI markers of CSVD. APOE ε4 carrier status and APOE ε4 genotype were associated with increasing WMH burden and the presence of CMBs, especially lobar, while APOE ε2 carrier status was associated with increasing WMH load [41]. It has also been reported that high LDL cholesterol levels are associated with reduced WMH volume, and Lp(a) may share similar features with LDL cholesterol since Lp(a) and LDL both contain apoB100 [42].

Our study has several limitations. First of all, the sample size of our study is relatively small, the time span of patient collection is long, and inconsistent batches of reagents used for testing may lead to poor uniformity of testing. Secondly, this study is a cross-sectional study, which cannot show the impact of Lp(a) on the progression of small vascular disease nor the correlation between serum Lp(a) level and AD progress; thus, prospective large-sample studies are needed to confirm this relationship. Thirdly, APOE was not examined in the AD population, and the effect of Lp(a) on CSVD in different APOE populations could not be distinguished.

## 5. Conclusions

There is a negative correlation between serum Lp(a) levels and the occurrence and burden of CSVD in the AD population, especially CSO-EPVS and CMB. A high serum Lp(a) level may have a protective effect on the risk of CVSD in AD patients. The mechanism is still unclear and further study is needed.

## Figures and Tables

**Table 1:** Baseline demographic and clinical characteristics according to tertiles of Lp(a) level.

Variables	Total (n = 111)	Tertiles of Lp(a) Level			*p* Value
		T1 (n = 37)	T2 (n = 37)	T3 (n = 37)	
Demographic data					
Age, means ± SD	75.27 ± 9.70	75.46 ± 8.12	76.57 ± 9.83	73.78 ± 10.86	0.467
Male, n (%)	44 (39.6)	16 (43.2)	13 (35.1)	15 (40.5)	0.768
Current smoking, n (%)	49 (44.1)	16 (43.2)	17 (45.9)	16 (43.2)	0.964
Alcohol drinkers, n (%)	22 (19.8)	9 (24.3)	8 (21.6)	5(13.5)	0.479
BMI, kg/m^2^, means ± SD	23.5 ± 3.2	23.0 ± 2.9	23.9 ± 3.0	23.6 ± 3.4	0.175
FPG, mmol/L, means ± SD	5.55 ± 1.30	5.55 ± 1.17	5.71 ± 1/37	5.40 ± 1.35	0.606
TC, mmol/L, means ± SD	4.63 ± 3.95	4.11 ± 1.31	5.28 ± 6.58	4.50 ± 1.24	0.437
TG, mmol/L, means ± SD	1.41 ± 1.14	1.66 ± 1.72	1.30 ± 0.72	1.30 ± 0.60	0.289
LDL-C, mmol/L, means ± SD	2.48 ± 0.96	2.27 ± 0.96	2.41 ± 0.76	2.76 ± 1.08	0.076
HDL-C, mmol/L, means ± SD	1.16 ± 0.36	1.09 ± 0.32	1.23 ± 0.44	1.16 ± 0.32	0.282
BUN, mmol/L, means ± SD	6.46 ± 3.73	7.08 ± 4.75	5.87 ± 3.56	6.41 ± 2.46	0.141
Scr, umol/L, means ± SD	78.60 ± 44.42	81.65 ± 65.61	78.61 ± 28.51	75.53 ± 28.95	0.706
HbA1c (%), means ± SD	6.23 ± 1.06	6.28 ± 0.89	6.08 ± 1.18	5.95 ± 0.77	0.375
Medical history, n (%)					
Coronary heart disease	55 (49.5)	19 (51.3)	15 (40.5)	21 (56.8)	0.510
Atrial fibrillation	10 (9.0)	3 (8.1)	5 (13.5)	2 (5.4)	0.463
Hypertension	54 (48.6)	17 (45.9)	21 (56.8)	16 (43.2)	0.469
Diabetes	40 (36.0)	12 (32.4)	15 (40.5)	13 (35.1)	0.761
Dyslipidemia	22 (19.8)	7 (18.9)	7 (18.9)	8 (21.6)	0.945
Imaging markers, n (%)					
Cerebral small vessel diseases	89 (80.2)	35 (94.6)	29 (78.4)	25 (67.6)	0.013
BG-EPVS > 10	77 (69.4)	30 (81.1)	25 (67.6)	22 (59.5)	0.125
CSO-EPVS > 10	85 (76.6)	36 (97.3)	27 (73.0)	22 (59.5)	0.001
Lacunes	47 (42.3)	20 (54.1)	15 (40.5)	12 (32.4)	0.164
Confluent WMH	41 (36.9)	15 (40.5)	16 (43.2)	10 (27.0)	0.302
CMBs	21 (18.9)	10 (27.0)	8 (21.6)	3 (8.1)	0.111

*p* value tests difference of baseline characteristics among Lp(a) tertile groups by analysis of variance for continuous variables and χ2 test for categorical variables. Tertiles of Lp(a): T1, <86 mg/L; T2, 86–183 mg/L; T3, ≥183 mg/L. Abbreviations: T, tertile; BMI, body mass index; FPG, fasting plasma glucose; TC, total cholesterol; TG, triglyceride; HDL-C, high-density lipoprotein cholesterol; LDL-C, low-density lipoprotein cholesterol; Lp(a), lipoprotein(a); BUN, blood urea nitrogen; Scr, serum creatinine; BG-EPVS, basal ganglia-enlarged perivascular spaces; WMH, white-matter hyperintensity; CSO-EPVS, centrum semiovale-enlarged perivascular spaces; CMBs, cerebral microbleeds.

**Table 2:** Odds Ratio for Presence or Absence of CSVD According to the grade of Lp(a).

Outcome	Lp(a) Categories	CSVD (n%)	Unadjusted cOR (95% CI)	*p* Value	Model 1 *		Model 2 †	
					Adjusted cOR (95% CI)	*p* Value	Adjusted cOR (95% CI)	*p* Value
Total CSVD Score ‡	T1	35 (94.6)	Ref		Ref		Ref	
	T2	29 (78.4)	0.207 (0.041–1.053)	0.058	0.141 (0.023–0.841)	0.032	0.132 (0.018–0.946)	0.044
	T3	25 (66.2)	0.119 (0.024–0.579)	0.008	0.116 (0.021–0.655)	0.015	0.109 (0.016–0.737)	0.023

cOR indicates common odds ratio; CSVD, cerebral small vessel disease. * Model 1: adjusted for age and sex. † Model 2: adjusted for age, sex, BMI, current smoker, current drinker, hypertension, diabetes, history of atrial fibrillation and coronary heart disease, TC, TG, HDL-C, LDL-C. ‡ Total CSVD score: one point allocated for presence of lacunes, microbleeds, moderate-to-severe (>10) PVS in basal ganglia, periventricular WMH Fazekas 3, or deep WMH Fazekas 2–3. Presence of CSVD was defined as patient with a total CSVD score ≥ 1 point.

**Table 3:** Ordinal Logistic Regression Analysis for the Association of Lp(a) level With Total CSVD Score.

Outcome	Lp(a) Category	Unadjusted cOR (95% CI)	*p* Value	Model 1 *		Model 2 †	
				Adjusted cOR (95% CI)	*p* Value	Adjusted cOR (95% CI)	*p* Value
Total CSVD score ‡	T1	Ref		Ref		Ref	
	T2	0.743 (0.431–1.279)	0.284	0.696 (0.427–1.134)	0.145	0.722 (0.452–1.154)	0.174
	T3	0.457 (0.265–0.787)	0.005	0.501 (0.308–0.815)	0.005	0.576 (0.362–0. 915)	0.019

cOR indicates common odds ratio; CSVD, cerebral small vessel disease. * Model 1: adjusted for age and sex. † Model 2: adjusted for age, sex, BMI, current smoker, current drinker, hypertension, diabetes, history of atrial fibrillation and coronary heart disease, TC, TG, HDL-C, LDL-C. ‡ Total CSVD score: 1 point allocated for presence of lacunes, microbleeds, moderate-to-severe (>10) PVS in basal ganglia, periventricular WMH Fazekas 3, or deep WMH Fazekas 2–3.

**Table 4:** The association between Lp(a) level and CSVD MRI markers.

Outcome	Lp(a) Category	Unadjusted cOR (95%CI)	*p* Value	Model 1 *		Model 2 †	
				Adjusted cOR (95% CI)	*p* Value	Adjusted cOR (95% CI)	*p* Value
BG-EPVS > 10 (moderate-to-severe) and	T1	ref		ref		ref	
	T2	0.486 (0.166–1.421)	0.188	0.377 (0.780–1.231)	0.115	0.582 (0.154–2.200)	0.425
	T3	0.342 (0.119–0.980)	0.046	0.366 (0.113–1.091)	0.095	0.530 (0.143–1.964)	0.342
CSO-EPVS >10 (moderate-to-severe) and	T1	ref		ref		ref	
	T2	0.075 (0.009–0.622)	0.016	0.070 (0.008–0.583)	0.014	0.059 (0.006–0.542)	0.012
	T3	0.041 (0.005–0.330)	0.003	0.041 (0.005–0.583)	0.003	0.029 (0.003–0.273)	0.002
Presence of Lacunes	T1	ref		ref		ref	
	T2	0.580 (0.231–1.456)	0.246	0.439 (0.159–1.217)	0.114	0.636 (0.168–2.407)	0.505
	T3	0.408 (0.159–1.049)	0.063	0.383 (0.135–1.090)	0.072	0.536 (0.149–1.928)	0.340
Periventricular WMH	T1	ref		ref		ref	
	T2	1.135 (0.424–3.039)	0.802	0.976 (0.344–2.767)	0.964	0.869 (0.270–2.799)	0.814
	T3	0.457 (0.149–1.406)	0.172	0.437 (0.133–1.431)	0.171	0.496 (0.140–1.760)	0.278
Deep WMH	T1	ref		ref		ref	
	T2	0.704 (0.272–1.822)	0.469	0.635 (0.240–1.684)	0.362	0.594 (0.206–1.711)	0.334
	T3	0.471 (0.174–1.278)	0.139	0.473 (0.171–1.312)	0.150	0.505 (0.173–1.472)	0.211
Confluent WMH #	T1	ref		ref		ref	
	T2	1.117 (0.444–2.815)	0.814	0.903 (0.386–2.608)	0.995	0.940 (0.384–3.116)	0.867
	T3	0.543 (0.204–1.445)	0.221	0.542 (0.197–1.493)	0.236	0.673 (0.230–1.971)	0.470
Presence of CMBs ‡	T1	ref		ref		ref	
	T2	0.745 (0.350–1.584)	0.588	0.802 (0.268–2.395)	0.692	0.652 (0.193–2.205)	0.491
	T3	0.238 (0.089–0.635)	0.042	0.233 (0.057–0.935)	0.043	0.144 (0.029–0.716)	0.018

cOR indicates common odds ratio; CSVD, cerebral small vessel disease. * Model 1: adjusted for age and sex. † Model 2: adjusted for age, sex, BMI, current smoker, current drinker, hypertension, diabetes, history of atrial fibrillation and coronary heart disease, TC, TG, HDL-C, LDL-C. # confluent WMH was defined as either (early) confluent deep WMH (Fazekas score 2 or 3) or irregular periventricular WMH extending into the deep white matter (Fazekas score 3); ‡ Presence of cerebral microbleeds (CMBs) was defined as presence of any CMBs; and BG-EPVS (moderate-to-severe) indicated moderate-to-severe (>10) perivascular spaces in basal ganglia; andCSO-EPVS (moderate-to-severe) indicated moderate-to-severe (>10) perivascular spaces in centrum semiovale.

## Data Availability

The data presented in this study are available from the corresponding authors upon reasonable request. The data are not publicly available due to privacy reasons.
